# Shift of cell-death mechanisms in primary human neutrophils with a ruthenium photosensitizer

**DOI:** 10.1007/s00775-024-02088-4

**Published:** 2024-12-14

**Authors:** Nicolás Montesdeoca, Jennifer M. Mohr, Sebastian Kruss, Johannes Karges

**Affiliations:** 1 Faculty of Chemistry and Biochemistry, Ruhr-University Bochum, Universitätsstrasse 150, 44780 Bochum, Germany; 2 Fraunhofer Institute for Microelectronic Circuits and Systems, Duisburg, Germany

**Keywords:** Bioinorganic chemistry, Medicinal inorganic chemistry, Metals in medicine, NETosis, Primary human neutrophils

## Abstract

**Abstract:**

Primary human neutrophils are the most abundant human white blood cells and are central for innate immunity. They act as early responders at inflammation sites, guided by chemotactic gradients to find infection or inflammation sites. Neutrophils can undergo both apoptosis as well as NETosis. NETosis is a form of neutrophil cell death that releases chromatin-based extracellular traps (NETs) to capture and neutralize pathogens. Understanding or controlling the balance between these cell-death mechanisms is crucial. In this study, the chemical synthesis and biologic assessment of a ruthenium complex as a light-activated photosensitizer that creates reactive oxygen species (ROS) in primary human neutrophils is reported. The ruthenium complex remains non-toxic in the dark. However, upon exposure to blue light at 450 nm, it exhibits potent cytotoxic effects in both cancerous and non-cancerous cell lines. Interestingly, the metal complex shifts the cell-death mechanism of primary human neutrophils from NETosis to apoptosis. Cells irradiated directly by the light source immediately undergo apoptosis, whereas those further away from the light source perform NETosis at a slower rate. This indicates that high ROS levels trigger apoptosis and lower ROS levels NETosis. The ability to control the type of cell death undergone in primary human neutrophils could have implications in managing acute and chronic infectious diseases.

**Graphical abstract:**

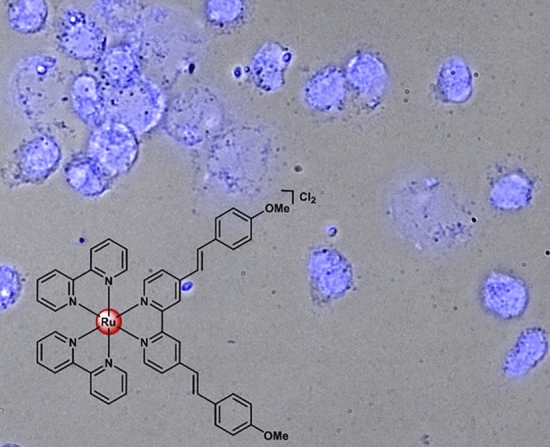

**Supplementary Information:**

The online version contains supplementary material available at 10.1007/s00775-024-02088-4.

## Introduction

Light-activated therapeutics are receiving increasing attention in the chemical and biologic literature due to spatial and temporal control over the treatment. One of the most promising therapeutic techniques is photodynamic therapy (PDT) [1, 2]. During PDT, a photosensitizer is locally or systemically administrated into the body. Although initially non-specifically distributed throughout the body, in the ideal scenario the photosensitizer accumulates in the targeted tissue after a specific incubation time. The incubation time can vary depending on the specific photosensitizer and the procedure, typically ranging from 5 min to 24 h. Following this incubation period, the targeted tissue is exposed to light irradiation, causing the localized and catalytic generation of reactive oxygen species (ROS). This oxidative stress ultimately results in cell death of the treated cells. It is important to note that the therapeutic effect occurs exclusively in the tissue containing the photosensitizer during irradiation, offering the potential for highly precise and selective treatment [3–9].

The majority of photosensitizers studied in the chemical literature as well as used inside the clinics are based on a tetrapyrrolic structural core (e.g., porphyrin, chlorin, bacteriochlorin, phthalocyanine), which significantly influences their photophysical and biologic characteristics. Leveraging this common structural feature, most of these photosensitizers exhibit similar limitations, including low water solubility, low photostability, tedious synthesis/purification procedures, slow clearance from the body leading to photosensitivity, and poor cancer selectivity [10–14]. To address some of these challenges, research endeavors have focused on integrating metal ions. Certain metals have the capability to enhance the molecule’s stability against metabolic processes and light exposure. Moreover, specific heavy atoms may positively influence intersystem crossing efficiency through the heavy atom effect, thereby enhancing ROS generation. To date, there has been growing interest in ruthenium(II) polypyridine complexes due to their appealing photophysical and biologic properties (i.e., strong luminescence, long luminescence lifetime, high water solubility, efficient cellular uptake, high (photo-)stability) [15–22]. It is important to highlight that the ruthenium(II) polypyridine complex TLD-1433 has advanced into clinical trials in Canada for the treatment of non-muscle invasive bladder cancer [23].

Based on the ability to generate unselectively oxidative stress inside the cancerous cell when exposed to irradiation, the cell-death mechanism can depend on various factors such as the specific photosensitizer, subcellular distribution of the photosensitizer, or cell line. While previous studies have demonstrated that photosensitizers can induce necroptosis, paraptosis, pyroptosis, autophagy, immunogenic cell death, or ferroptosis, the vast majority of compounds trigger apoptosis or necrosis. Notably, photosensitizers can also interact through multimodal cell-death mechanisms [24–27].

Neutrophilic granulocytes (neutrophils) make up 40–70% of all human white blood cells and play an essential role in the innate immune system [28]. They are among the first responders at inflammatory sites and attracted by chemotactic gradients that develop around such sites—a process called chemotaxis [29]. This involves the migration through both blood vessels and interstitial space. At the site of infection, neutrophils release cytokines and thereby attract other immune cells such as macrophages, which amplifies the overall inflammatory reaction [30]. In addition to recruiting other immune cells, neutrophils are essential for directly combating microorganisms through mechanisms such as phagocytosis, degranulation, and the formation of neutrophil extracellular traps (NETs) via the process of NETosis [31, 32]. Neutrophils can perform both apoptosis or NETosis. During NETosis the chromatin inside the nucleus expands via entropic swelling until it ruptures both nuclear envelope and outer cell membrane [33, 34]. In addition, the neutrophils undergo a number of changes, e.g. a change in the cell surface markers that mark them for elimination by other phagocytic cells such as mast cells [35]. NETosis is regulated by many different factors including cell adhesion, small molecules or cytokines but can also be triggered by UV light [36–39]. Moreover, NETosis can be used for transport and release of cargo (nano)materials [40].

Phagocytosis of apoptotic and NETotic neutrophils is necessary for the ultimate resolution of the inflammation site. Therefore, inflammatory diseases can be treated by controlling apoptosis [41]. In addition, apoptosis can be up- or down-regulated by microorganisms depending on the health status of the host and the evasion strategies [42]. Neutrophil apoptosis aids in resolving inflammation, is crucial for pathogen clearance and therefore enhances immune response against infections. As a dysregulated neutrophil apoptosis contributes to autoimmune disorders like arthritis and Chronic obstructive pulmonary disease (COPD) this gives potential implications for managing acute and chronic infectious diseases [43, 44].

Herein, the biologic evaluation of a ruthenium complex as a light-activated photosensitizer that is able to change the cell-death mechanism of primary human neutrophils from NETosis to apoptosis is presented. While the ruthenium complex is non-toxic in the dark, it induces a strong therapeutic effect upon exposure to irradiation. Various cell lines (mouse colon carcinoma, human breast adenocarcinoma, human fibroblast cells) are used to study if the compound is able to selectively induce apoptosis upon irradiation with light. Based on these results cell viability and morphology of primary human neutrophils upon treatment with the metal complex is studied and the impact on apoptosis and NETosis is quantified.

## Results and discussion

The herein studied [Ru(2,2´-bipyridine)2((*E*,*E*’)-4,4´-Bis[p-(*N*,*N*-methoxy)styryl]-2,2´-bipyridine)][Cl]_2_ complex (**Ru**, Scheme S1) was prepared according to its original publication. The identity of **Ru** and its precursors was verified by nuclear magnetic resonance spectroscopy (Figs. S1–S7) and was found to be in agreement with the previous literature [45]. The photophysical properties, specifically absorption and emission, of **Ru** were also evaluated (Figs. S8–S9). Following, the cytotoxicity of **Ru** in comparison to cisplatin in the dark as well as upon irradiation (450 nm, power: 20%, 10 min, 1.2 J/cm^2^) was assessed in cancerous mouse colon carcinoma (CT-26), human breast adenocarcinoma (MCF-7), and non-cancerous human fibroblast (GM-5657) cells using the dye 3-(4,5-dimethylthiazol-2-yl)-2,5-diphenyltetrazolium bromide (MTT). Cisplatin, used as the control substance, was found to be cytotoxic in the low micromolar range in all evaluated cell lines. In contrast, the ruthenium(II) polypyridine complex **Ru** demonstrated to be non-toxic in the dark (IC_50,dark_ > 100 μM) but phototoxic upon exposure to irradiation in the very low micromolar range (IC_50,light_ = 1.1 – 1.5 μM) against all cell lines (Table 1).

**Table 1:** (Photo-)toxicity of **Ru** in comparison to cisplatin in the dark as well as upon irradiation (450 nm, power: 20%, 10 min, 1.2 J/cm^2^) in cancerous mouse colon carcinoma (CT-26), human breast adenocarcinoma (MCF-7), and non-cancerous human fibroblast (GM-5657) cells using the dye 3-(4,5-dimethylthiazol-2-yl)-2,5-diphenyltetrazolium bromide (MTT)

	Ru		Cisplatin	
	Dark	Light	Dark	Light
MCF-7	> 100	1.1 ± 0.2	8.3 ± 0.2	8.1 ± 0.2
CT-26	> 100	1.5 ± 0.3	6.0 ± 0.3	5.8 ± 0.5
GM-5657	> 100	1.4 ± 0.3	8.3 ± 0.8	8.7 ± 0.4

The error bars correspond to the standard deviation of three independent measurements (mean ± SD)

**Fig. 1:**
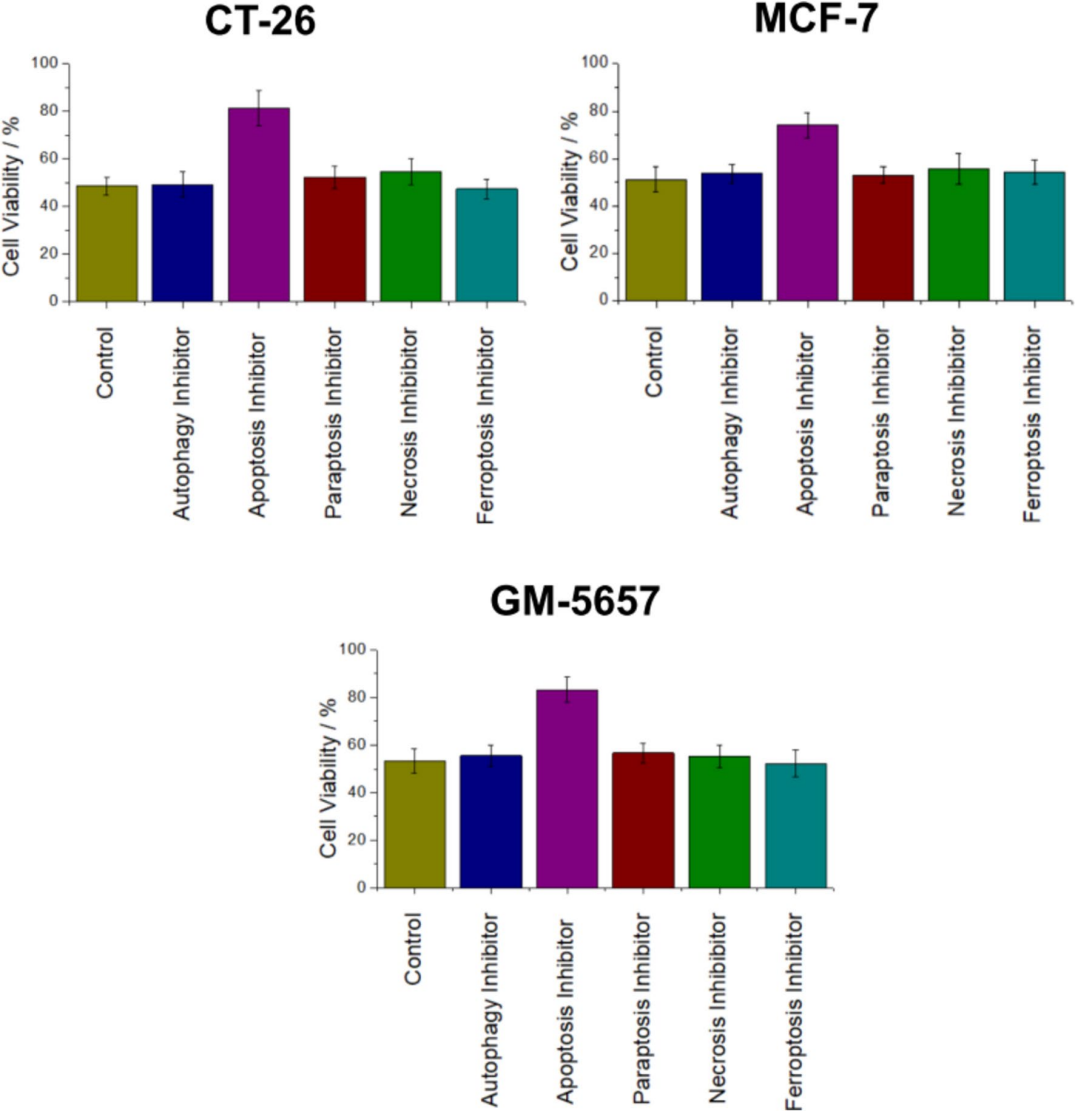
Identification of the type of cell death generated by **Ru** in CT-26, MCF-7, and GM-5657 cells. Quantification of the cell viability upon pre-incubation with autophagy (100 μM, 3-methyladenine), apoptosis (20 μM, Z-VAD-FMK), paraptosis (0.1 μM, cycloheximide), necrosis (60 μM, necrostatin-1), ferroptosis (50 μM, ferrostatin-1) inhibitors, or an iron chelator (100 μM, deferoxamine), that are rendering the ability of the metal complex to induce the specific cell-death mechanism, treated with the IC_50_ value of the photosensitizer in the light (CT-26: 1.5 μM, MCF-7: 1.1 μM, GM-5657: 1.4 μM) and exposed to irradiation (450 nm, power: 20%, 10 min, 1.2 J/cm^2^), and the cell viability determined using a MTT assay. The error bars correspond to the standard deviation. *n* = 3

For an insight into the biologic effects caused by the photosensitizer, the cell-death mechanism was studied. The cells were pre-incubated with autophagy (3-methyladenine), paraptosis (cycloheximide), necrosis (necrostatin-1), apoptosis (Z-VAD-FMK), or ferroptosis (ferrostatin-1) inhibitors, that are rendering the ability of the photosensitizer to trigger this cell-death mechanism, treated with the corresponding **Ru** IC_50_ value upon irradiation, and exposed to irradiation, and the cell viability determined using a MTT assay [46]. Interestingly, the metal complex showed the same trends in CT-26, MCF-7, and GM-5657 cell lines. While the pre-incubation with autophagy, paraptosis, necrosis or ferroptosis inhibitors did not influence the cell survival, the pre-incubation with apoptosis inhibitors did strongly elevate the cell viability (Fig. 1). These findings suggest that the photosensitizer specifically induces apoptosis.

**Fig. 2:**
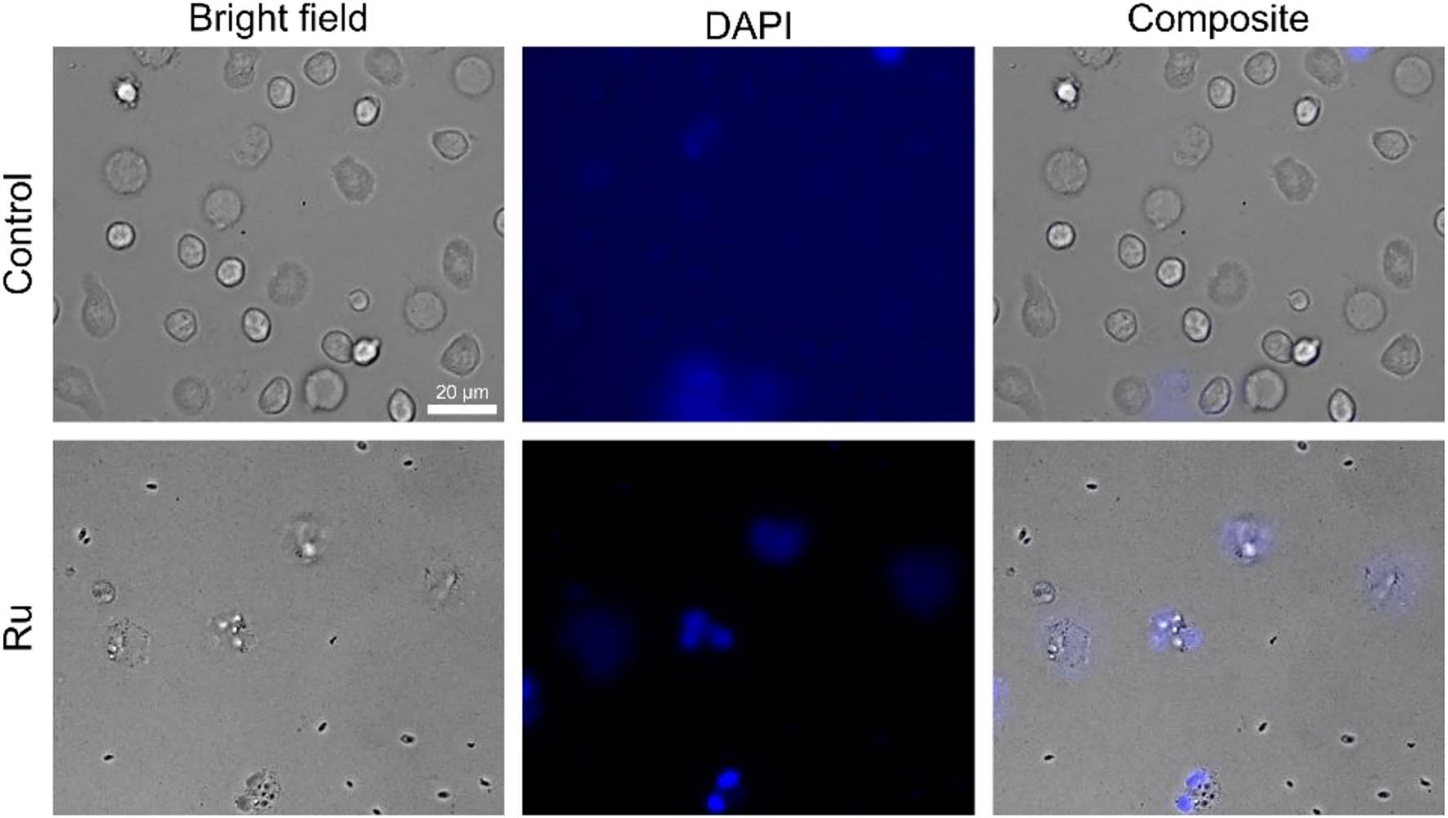
Response of neutrophilic granulocytes to **Ru** after irradiation with a LED at 555 nm with 200 mW. Human neutrophilic granulocytes are shown in a bright field image, a fluorescence image after staining with DAPI and a composite with and without Ru/light exposure. Cells treated with **Ru** are all apoptotic (dye can enter the cell), control cells are mostly alive

To gain further insight into the biologic effects of **Ru**, freshly isolated primary human neutrophil granulocytes were incubated with the complex and irradiated with a LED at 555 nm with 200 mW, the results were compared with a control group without **Ru**. The cell response was evaluated 2 h after irradiation (Fig. 2) by a DAPI staining that should only enter dead cells. In addition, NETosis can be evaluated by the morphology of the cell nucleus. NETosis is characterized by chromatin swelling and a distributed DNA in and around the cells [39]. In contrast, in apoptosis the nucleus stays lobulated and condensed.

**Fig. 3:**
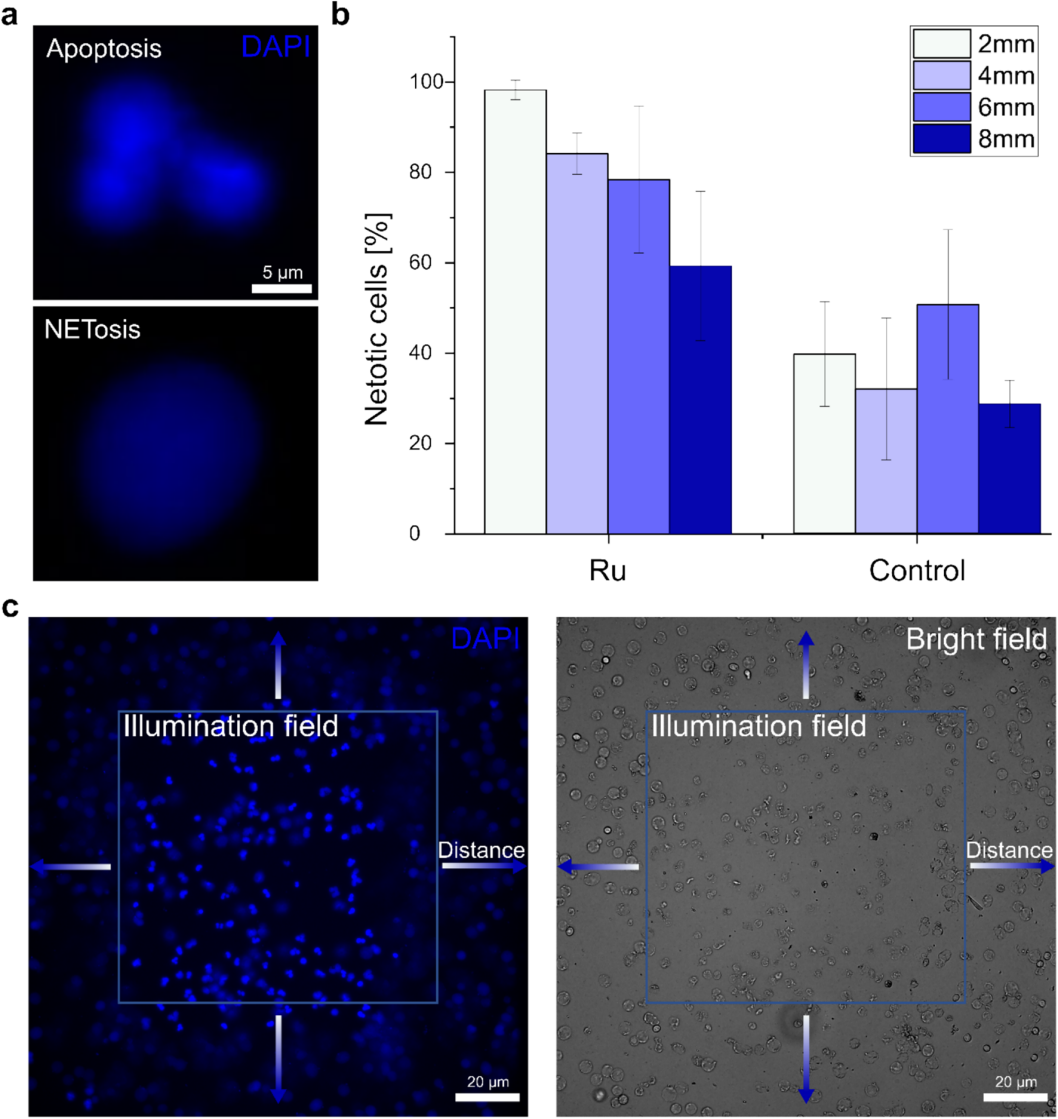
Distance dependent NETosis. **a** Exemplary apoptotic and NETotic human neutrophilic granulocytes stained with DAPI. **b** Illumination field and adjacent area of fluorescence and bright field images. Neutrophilic granulocytes within the illumination field are apoptotic after irradiation with a LED at 555 nm with 200 mW for 10 min whereas cells around this region show distance-dependent NETosis. **c** Percentage of NETosis of human neutrophilic granulocytes related to their distance from the illumination field ranging from 2 to 8 mm with and without incubation of **Ru.** Error bars = SD (*n* = 12 with 3 donors). The highest percentage is closest to the illumination field and the lowest is farest away with **Ru**. The control experiments do not show a clear distance dependence

Within the illumination field, the cells with the photosensitizer were apoptotic, surrounded by an almost clear line around the field of irradiation in the immediate vicinity where cells were NETotic (Fig. 3a and b). With increasing distance from the illumination field, the proportion of NETosis decreased (Fig. 3c). Here, the term distance represents the spatial separation from the center of the illumination field. A series of images were captured at increasing distance intervals of 2 mm, 4 mm, 6 mm, and 8 mm from the source upwards, downwards and to the right and left. These results underline the hypothesis that the photosensitizer specifically triggers apoptosis under direct illumination. In contrast, smaller ROS levels or other molecules released by the apoptotic cells trigger NETosis in the nearby vicinity. Previous studies have shown that ROS are essential for NETosis as they activate pathways that lead to NETosis, such as the degradation of chromatin or the oxidation of DNA. This is reflected in the inhibition of ROS production, which interrupts the process [47, 48]. A high dependence on the illumination distance was observed during this process, in which the highest NETosis rates were obtained in the closest proximity to the illumination area (2 mm). The amount of NETotic cells decreased with increasing distance from the illumination field (8 mm).

**Fig. 4:**
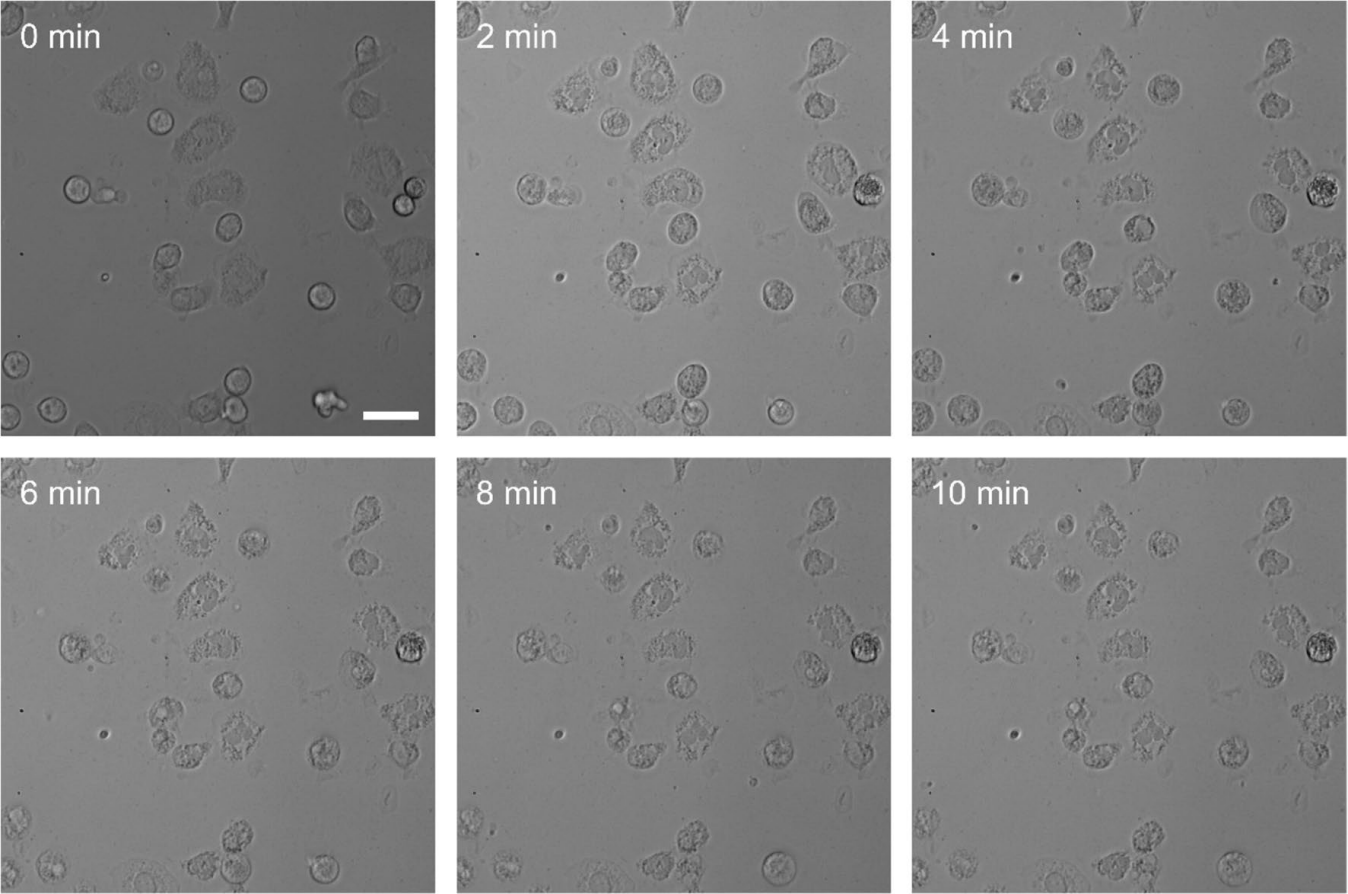
Time course during irradiation with 200 mW at 555 nm for 10 min of neutrophilic granulocytes which were prior incubated with **Ru**

For an insight into the time-dependent induction of cell death, the morphology of the neutrophilic granulocytes was monitored upon treatment with **Ru** and irradiation. Within several minutes, clear morphologic changes were observed indicative for the rapid photo-induced triggering of apoptosis (Fig. 4). Note that compaction of chromatin is a clear morphological sign of apoptosis whereas chromatin expansion corresponds to NETosis [32, 33]. It is important to emphasize the morphologic changes in such a short time. Combined these results show that **Ru** upon irradiation triggers apoptosis and in the vicinity NETosis. This can be explained by the direct exposure of neutrophils in the illumination field to ROS species generated by **Ru** upon irradiation, along with the diffusion of signaling molecules from apoptotic cells, which induce NETosis in the surrounding cells. Therefore, it can be used to tune the ratio between apoptosis and NETosis, which can be used for tailored medical treatments in the future. NETosis is not only a process that happens in response to pathogens. More importantly, it is highly relevant in any inflammation given the fact that neutrophils are the most abundant type of immune cell present in all tissues. In this context NETosis can have both ‘positive’ or ‘negative’ consequences for diseases such as autoimmune diseases as well as e.g. cancer metastasis depending on the immunological context [36, 49]. Therefore, controlling the level or rate of NETosis, particularly through the use of light, represents a promising therapeutic target.

## Conclusions

In this study, the chemical synthesis and biologic assessment of a ruthenium complex photosensitizer capable of shifting the cell-death mechanism of primary human neutrophils from NETosis to apoptosis is reported. While the ruthenium complex remains non-toxic in the absence of light, it exhibits a potent therapeutic effect when exposed to irradiation. Testing across different cell lines indicates that the compound can selectively induce apoptosis upon light irradiation. The biologic evaluation of the ruthenium complex in human neutrophils suggested the triggering of apoptosis and NETosis within these cells with a high dependence on the illumination distance regarding NETosis. Surprisingly within the illumination field predominantly apoptosis of neutrophils occurred while more cells underwent NETosis in close vicinity. In addition, the rapid start of apoptosis within 10 min in the illumination field is intriguing, showing the potential of **Ru**. The ability to control the type of cell death undergone in primary human neutrophils could have implications in managing acute and chronic infections and inflammatory diseases. Further studies are required to elucidate the mechanism of this process and to explore applications of this photosensitizer to tune inflammation.

## Supplementary Information

Below is the link to the electronic supplementary material.

Supplementary file1 (DOCX 574 KB)

## Data Availability

The data that support the findings of this study are available in the manuscript or supplementary material of this article.
