# Supplementary material for: Shift of cell-death mechanisms in primary human neutrophils with a ruthenium photosensitizer
Source: J Biol Inorg Chem. 2024 Dec 14;30(1):53–60. doi: 10.1007/s00775-024-02088-4 (PMC11914334; doi:10.1007/s00775-024-02088-4)
Supplement: Supplementary file 1 — Supplementary file1 (DOCX 574 KB) [file 775_2024_2088_MOESM1_ESM.docx]

Supporting Information:

**Shift of Cell Death Mechanisms in Primary Human Neutrophils with a Ruthenium Photosensitizer**

*Nicolás Montesdeoca ^a,#^, Jennifer Mohr ^a,#^, Sebastian Kruss ^a,b,^*, Johannes Karges^a,^**

^a^ Faculty of Chemistry and Biochemistry, Ruhr-University Bochum, Universitätsstrasse 150, 44780 Bochum, Germany.

^b^ Fraunhofer Institute for Microelectronic circuits and systems

* Corresponding author: Email: sebastian.kruss@ruhr-uni-bochum.de; Email: johannes.karges@ruhr-uni-bochum.de, Tel: +49 2343224187; WWW: www.kargesgroup.ruhr-uni-bochum.de

TABLE OF CONTENTS

[EXPERIMENTAL SECTION 3](#_Toc181869773)

[Materials 3](#_Toc181869774)

[Instrumentation and Methods 3](#_Toc181869775)

[Synthesis 3](#_Toc181869776)

[[Ru(DMSO)_4_(Cl)_2_] 3](#_Toc181869777)

[[Ru(2,2′-bipyridine)_2_(Cl)_2_] 4](#_Toc181869778)

[(E,E′)-4,4′-Bis[p-methoxystyryl]-2,2′-bipyridine 4](#_Toc181869779)

[[Ru(2,2′-bipyridine)_2_((E,E′)-4,4′-Bis[p-methoxystyryl]-2,2′-bipyridine)][Cl]_2_ (**Ru**) 5](#_Toc181869780)

[Measurement of Absorption Spectra 5](#_Toc181869781)

[Measurement of Emission Spectra 6](#_Toc181869782)

[Cell Culture 6](#_Toc181869783)

[(Photo-)toxicity against Cancer Cells 6](#_Toc181869784)

[Cell Death Mechanism 7](#_Toc181869785)

[Isolation of neutrophilic granulocytes 7](#_Toc181869786)

[Induction of cell death in neutrophilic granulocytes 8](#_Toc181869787)

[SUPPORTING FIGURES 9](#_Toc181869788)

[Scheme S1. Synthetic strategy for the preparation of [Ru(2,2´-bipyridine)_2_((E,E’)-4,4´-Bis[p-(N,N-methoxy)styryl]-2,2´-bipyridine)][Cl]_2_ (**Ru**). 9](#_Toc181869789)

[Figure S1. ^1^H-NMR spectrum (400 mhz) of [Ru(DMSO)_4_(Cl)_2_] in D_2_O. 10](#_Toc181869790)

[Figure S2. ^13^C-NMR spectrum (100 mhz) of [Ru(DMSO)_4_(Cl)_2_] in D_2_O. 11](#_Toc181869791)

[Figure S3. ^1^H-NMR spectrum (400 mhz) of [Ru(2,2′-bipyridine)_2_(Cl)_2_] in DMSO-d_6_. 12](#_Toc181869792)

[Figure S4. ^13^C-NMR spectrum (100 mhz) of [Ru(2,2′-bipyridine)_2_(Cl)_2_] in DMSO-d_6_. 13](#_Toc181869793)

[Figure S5. ^1^H-NMR spectrum (400 mhz) of (E,E′)-4,4′-Bis[p-methoxystyryl]-2,2′-bipyridine in DCM-d_2_. 14](#_Toc181869794)

[Figure S6. ^13^C-NMR spectrum (100 mhz) of (E,E′)-4,4′-Bis[p-methoxystyryl]-2,2′-bipyridine in DCM-d_2_. 15](#_Toc181869795)

[Figure S7. ^1^H-NMR spectrum (400 mhz) of [Ru(2,2′-bipyridine)_2_((E,E′)-4,4′-Bis[p-methoxystyryl]-2,2′-bipyridine)][Cl]_2_ in ACN-d_3_. 16](#_Toc181869796)

[Figure S8. Normalized absorption spectra of Ru in water. 17](#_Toc181869797)

[Figure S9. Normalized emission spectra of Ru in water. 18](#_Toc181869798)

# EXPERIMENTAL SECTION

## Materials

All chemicals were obtained from commercial sources and were used without further purification. All solvents purchased were of analytical or high-performance liquid chromatography (HPLC) grade. The solutions, media, or buffers employed were obtained from Gibco, ThermoFisher Scientific, or Signa-Aldrich and were used without any additional treatments, unless otherwise specified.

## Instrumentation and Methods

Schlenk glassware and a vacuum line were employed when reactions sensitive to moisture/oxygen had to be performed under a nitrogen atmosphere. Thin-layer chromatography (TLC) was performed using silica gel 60 F-254 (Merck) plates, with the detection of spots being achieved by exposure to UV light. ^1^H- and ^13^C-NMR spectra were recorded on a 400 MHz NMR spectrometer (Bruker). Chemical shifts (δ) are reported in parts per million (ppm) referenced to tetramethylsilane (δ 0.00) ppm using the residual proton solvent peaks as internal standards. Coupling constants (*J*) are reported in Hertz (Hz) and the multiplicity is abbreviated, as follows: s (singlet), d (doublet), t (triplet), dd (doublet of doublets), td (triplet of doublets), and m (multiplet).

## Synthesis

### [Ru(DMSO)_4_(Cl)_2_]

The compound was prepared using a similar procedure as previously reported (I. P. Evans, A. Spencer, G. Wilkinson *J. Chem. Soc. Dalton Trans.* **1973**, 204-209). Ruthenium(III)-chloride hydrate (2 g, 7,64 mmol, assuming that xH_2_O = 3) was partially dissolved in ethanol (50 mL) and refluxed for 3 hours protected from light. The compound dissolved completely, and the color changed from brown to deep green. The solvent was evaporated, and an oily green residue was obtained which was mix with dimethyl sulfoxide (8 mL). The mixture was heated at 150 °C for 2 hours protected from light. A color change from dark green to yellow was observed. Then the solution was cooled down to room temperature and acetone (100 ml) was added. The mixture was placed in the fridge overnight and the crude product precipitated as a bright yellow powder. The product was collected by filtration and washed with acetone (20 mL). To obtain more product, the filtrate was evaporated, acetone (50 mL) was added, and the mixture was placed in the fridge overnight. More product was obtained, collected, and washed as before. This procedure was repeated three times to yield the maximum amount of product. 3.48 g (7,1 mmol, 72 %) of [Ru(DMSO)_4_(Cl)_2_] were yielded as a bright yellow powder. ^1^H NMR (400 MHz, D_2_O): δ 3.52 (s, 6H), 3.50 (s, 6H), 3.42 (s, 6H), 2.75 (s, 6H). ^13^C NMR (101 MHz, D_2_O): δ 46.77, 46.55, 45.74, 45.15, 44.68, 44.36, 38.70.

### [Ru(2,2′-bipyridine)_2_(Cl)_2_]

The compound was prepared using a similar procedure as previously reported (C. E. McCusker, J. K. McKusker *Inorg. Chem.* **2011**, 50, 1656-1669). [Ru(DMSO)_4_Cl_2_] (1800 mg, 3.72 mmol, 1.00 eq.), 2,2´-bipyridine (1044 mg, 6.69 mmol, 1.8 eq.), and LiCl (14.18 g) were dissolved in dry *N*,*N*-dimethylformamide (50 ml) protected from light and under nitrogen atmosphere. The mixture was refluxed for 4 hours until the solution turned dark purple. Then the solution was cooled down to room temperature and acetone (500 ml) was added. The mixture was placed in the freezer overnight and the crude product precipitated. The product was collected by filtration and washed with distilled water (~100 ml) to remove the excess of LiCl and unwanted [Ru(bpy)_3_]^2+^. Finally, the product was washed with diethyl ether and dried under vacuum. A solid dark purple powder was obtained (1080 mg, 2.23 mmol, 60%). ^1^H NMR (400 MHz, DMSO): δ 9.97 (d, *J* = 4.1 Hz, 2H), 8.63 (d, *J* = 8.1 Hz, 2H), 8.48 (d, *J* = 7.1 Hz, 2H), 8.06 (t, *J* = 7.8 Hz, 2H), 7.76 (t, *J* = 6.6 Hz, 2H), 7.67 (t, *J* = 7.8 Hz, 2H), 7.51 (d, *J* = 4.5 Hz, 2H), 7.10 (t, *J* = 6.6 Hz, 2H). ^13^C NMR (101 MHz, DMSO): δ 160.20, 158.19, 153.17, 151.97, 134.57, 133.32, 125.34, 125.25, 122.84, 122.50.

### (*E,E*′*)-*4,4′-Bis[p-methoxystyryl]-2,2′-bipyridine

The compound was prepared using a similar procedure as previously reported (J. Karges, S. Kuang, F. Maschietto, O. Blacque, I. Ciofini, H. Chao, G. Gasser *Nat. Commun.* **2020**, 11, 3262). 4,4′-Dimethyl-2,2′-bipyridine (1064 mg, 5.78 mmol, 1.0 equiv.) was dissolved in dry *N*,*N*-dimethylformamide (50 mL) under nitrogen atmosphere and 4-methoxybenzaldehyde (1.78 mL, 14.44 mmol, 2.5 equiv.) was added to the solution. Afterwards potassium *tert*-butoxide (5184 mg, 46.20 mmol, 8 equiv.) was added slowly. The color of the solution turned to green, and the mixture was stirred for 24 h. After that the mixture which turned bright was poured into water (400 mL) and the suspension cooled down to 5 °C. The obtained precipitate was collected by filtration and washed with methanol. The product was purified by recrystallization from boiling acetic acid. The obtained solid was dissolved in dichloromethane and the mixture was washed with a 5% aqueous lithium chloride solution, brine, and water. The solvent was removed, and the product was isolated by recrystallization from boiling acetic acid. 1958 mg of (*E*,*E*′)-4,4′-Bis[*p*-methoxystyryl]-2,2′-bipyridine (4.66 mmol, 81%) were yielded as a beige solid. ^1^H NMR (400 MHz, CD_2_Cl_2_): δ 8.62 (d, *J* = 5.1 Hz, 2H), 8.56 (d, *J* = 1.7 Hz, 2H), 7.55 (d, *J* = 8.7 Hz, 4H), 7.44 (d, *J* = 16.3 Hz, 2H), 7.40 (dd, *J* = 5.1, 1.8 Hz, 2H), 7.05 (d, *J* = 16.3 Hz, 2H), 6.95 (d, *J* = 8.8 Hz, 4H), 3.84 (s, 6H). ^13^C NMR (101 MHz, CD_2_Cl_2_): δ 160.66, 149.85, 146.37, 133.07, 130.00, 129.48, 128.80, 124.38, 121.07, 118.23, 114.66, 55.74.

### [Ru(2,2′-bipyridine)_2_((*E,E′)-*4,4′-Bis[p-methoxystyryl]-2,2′-bipyridine)][Cl]_2_ (Ru)

The compound was prepared using a similar procedure as previously reported (J. Karges, S. Kuang, F. Maschietto, O. Blacque, I. Ciofini, H. Chao, G. Gasser *Nat. Commun.* **2020**, 11, 3262). Ru(2,2′-bipyridine)_2_Cl_2_ hydrate (1000 mg, 2.065 mmol, 1.0 equiv.) and (*E*,*E*′)-4,4′-Bis[*p*-methoxystyryl]-2,2′-bipyridine (1.042 g, 2.48 mmol, 1.2 equiv.) were suspended in dry ethanol (250 mL) under nitrogen atmosphere and the mixture was refluxed for 6 h. Then the solution was cooled down and a saturated aqueous solution of ammonium hexafluorophosphate was added. The crude product, which precipitated as a hexafluorophosphate salt was collected by filtration and washed with water and diethyl ether. The product was isolated via fractionated precipitation from acetonitrile by adding dropwise diethyl ether. The counter ion hexafluorophosphate was exchanged to chloride by elution with methanol from the ion exchange resin Amberlite IRA-410. 850 mg of **Ru** (1.02 mmol, 49%) were yielded as a dark red solid. ^1^H NMR (400 MHz, CD_3_CN): δ 8.65 (d, *J* = 1.9 Hz, 2H), 8.48 (d, *J* = 8.1 Hz, 4H), 8.03 (td, *J* = 7.9, 1.5 Hz, 4H), 7.82 (dd, *J* = 5.7, 1.5 Hz, 2H), 7.72 (dd, *J* = 5.6, 1.5 Hz, 2H), 7.68 (d, *J* = 16.5 Hz, 2H), 7.61 (d, *J* = 8.7 Hz, 4H), 7.57 (d, *J* = 5.9 Hz, 2H), 7.43 – 7.32 (m, 6H), 7.13 (d, *J* = 16.4 Hz, 2H), 7.00 (d, *J* = 8.8 Hz, 4H), 3.82 (s, 6H).

###

### Measurement of Absorption Spectra

For the absorption measurement, a 1 mM stock solution of **Ru** in acetonitrile was prepared. The measurement was conducted in water with a concentration of 30 μM of **Ru**, to keep the absorption intensity in the lambert-beer region. The used instrument was a Jasco V-670 Spectrometer.

### Measurement of Emission Spectra

For the emission measurement, a 1 mM stock solution of **Ru** in acetonitrile was prepared. The measurement was conducted in water with a concentration of 10 μM of **Ru**. The used slit width was 5 nm for both emission and excitation. The used instrument was a Jasco Spectrofluorometer FP-8300.

## Cell Culture

Murine mouse colon carcinoma cells (CT26) cells were cultured in RPMI 1640 medium supplemented with 10 % FBS Superior and 2 % penicillin/streptomycin. Human breast adenocarcinoma (MCF-7) cells were cultured in DMEM medium (4.5 g/L D-Glucose, L-Glutamine) supplemented with 10 % FBS Superior, 2.4 % penicillin/streptomycin, 1.2 % GlutaMAX-I and 1 % sodium pyruvate. Human fibroblasts (GM-5657) cells were cultured in DMEM medium (1 g/L D-Glucose) supplemented with 10 % FBS Superior, 2.4 % penicillin/streptomycin, 1.2 % GlutaMAX-I and 1 % sodium pyruvate. LGC Standards Ltd. provided all the cell lines, which were cultured at 37 °C with 10 % CO_2_. Prior to each experiment, the cells were passaged at least 3 times.

## (Photo-)toxicity against Cancer Cells

A total of 6 × 10^3^ cells were seeded onto 96-well plates and allowed to adhere overnight. The cells were treated with the compound which was diluted in cell media to a total volume of 200 μL. The cells were treated with increasing concentrations of the metal complex diluted in cell media achieving a total volume of 200 μL for 4 h at 37 °C and 10 % CO_2_. After this time, the drug supplemented media was removed and the cells incubated with fresh media. For the dark cytotoxicity, the cells were incubated in the dark inside the incubator for an additional 44 h at 37 °C and 10 % CO_2_. For the light cytotoxicity, the cells were irradiated (450 nm, power: 20%, 10 min, 1.2 J/cm^2^) and incubated in the dark inside the incubator for an additional 44 h at 37 °C and 10 % CO_2_. After the specified incubation period, the culture medium was substituted with phosphate-buffered saline buffer containing 3-(4,5-Dimethylthiazol-2-yl)-2,5-diphenyltetrazolium bromide (MTT) with a final concentration of 12 µM. The cells were incubated for 2 h at 37 °C and 10 % CO_2_ and the mixture was replaced by 200 µL DMSO. The concentration of the formazan dissolved in DMSO determined with an Infinite M Nano plus Microplate reader (Tecan).

## Cell Death Mechanism

A total of 6 × 10^3^ cells were seeded onto 96-well plates and allowed to adhere overnight. The cells were pre-incubated with 3-methyladenine (100 μM) as an autophagy inhibitor, Z-VAD-FMK (20 μM) as an apoptosis inhibitor, cycloheximide (0.1 μM) as a paraptosis inhibitor, necrostatin-1 (60 μM) as a necrosis inhibitor, or ferrostatin-1 (50 μM) as a ferroptosis inhibitor. The cells were treated with the IC_50_ value of the compound diluted in cell media achieving a total volume of 200 μL for 4 h at 37 °C and 10 % CO_2_. After this time, the drug supplemented media was removed and the cells incubated with fresh media. The cells were irradiated (450 nm, power: 20 %, 10 min, 1.2 J/cm^2^) and incubated in the dark inside the incubator for an additional 44  h at 37 °C and 10 % CO_2_. After the specified incubation period, the culture medium was substituted with phosphate-buffered saline buffer containing 3-(4,5-Dimethylthiazol-2-yl)-2,5-diphenyltetrazolium bromide (MTT) with a final concentration of 12 µM. The cells were incubated for 2 h at 37 °C and 10 % CO_2_ and the mixture was replaced by 200 µL DMSO. The concentration of the formazan dissolved in DMSO determined with an Infinite M Nano plus Microplate reader (Tecan).

## Isolation of neutrophilic granulocytes

Neutrophil experiments were performed in collaboration with Prof. Luise Erpenbeck. All experiments using human-derived cells were approved by the Ethics Committee Westfalen-Lippe (approval number 2021-657-f-S).  Neutrophils were obtained from fresh venous blood of healthy donors. All donors were fully informed about possible risks, and their informed consent was obtained in writing. The consent could be withdrawn at any time during the study. For the isolation of human neutrophilic granulocytes, the EasySep™ Direct Human Neutrophil Isolation Kit (Stemcell technologies, Canada) is used. In this process, 7.5 ml of human blood was collected into a sterile K3 EDTA S-Monovette® (Sarstedt, Germany) and allowed to cool to room temperature (RT). Next, 3-4 ml of the blood was transferred to a 15 ml falcon tube (Sarstedt, Germany). To this, 50 µl/ml of isolation cocktail and 50 µl/ml of rapid spheresTM were added, followed by a 5 min incubation at RT.

The sample was then brought to a total volume of 10 ml using PBS with 1 mM EDTA, inverted for mixing, and placed in a magnetic field for 5 min. The supernatant was transferred to a new falcon tube while in the magnet, with the same amount of rapid spheresTM added again, followed by another 5 min incubation outside the magnet and then in the magnet.

This process was repeated once more, with the supernatant being transferred to a new falcon tube while in the magnet. The isolated cells were then centrifuged for 3 min at 100 g, the supernatant was discarded, and the cell pellet was carefully resuspended in 1 ml of Roswell Park Memorial Institute (RPMI) medium 1640 for further use.

## Induction of cell death in neutrophilic granulocytes

A total of 6 × 10^5^ freshly isolated human neutrophilic granulocytes were seeded onto 35 mm glass dishes (MatTek, USA) and allowed to adhere for 30 min. The cells were incubated with the ruthenium complex (20 μM) in RPMI for 2 h at 37 °C and 5 % CO_2_. Control cells were also incubated in the same medium. Both ruthenium treated and control cells were irradiated with a LED light source at 555 nm at 200 mW for 10 min and the course of the cell death was imaged for 2 h using a square beam path. Afterwards the cells were stained with DAPI as a live dead staining to evaluate the percentage of apoptotic and netotic cells and they were imaged using a 395 nm LED.

# SUPPORTING FIGURES

Scheme S1. Synthetic strategy for the preparation of [Ru(2,2´-bipyridine)_2_((*E*,*E*’)-4,4´-Bis[p-(*N*,*N*-methoxy)styryl]-2,2´-bipyridine)][Cl]_2_ (Ru). a) EtOH, reflux, 3h; DMSO, 150 °C, 2h. b) 2,2´-bipyridine, LiCl, dry DMF, N_2_, reflux, 4h. c) dry DMF, N_2_; KOtBu, r.t., 24h. d) dry EtOH, N_2_, reflux, 6h; NH_4_PF_6_ (aq.); Amberlite IRA-410.

Figure S1. ^1^H-NMR spectrum (400 MHz) of [Ru(DMSO)_4_(Cl)_2_] in D_2_O.

Figure S2. ^13^C-NMR spectrum (100 MHz) of [Ru(DMSO)_4_(Cl)_2_] in D_2_O.

Figure S3. ^1^H-NMR spectrum (400 MHz) of [Ru(2,2′-bipyridine)_2_(Cl)_2_] in DMSO-d_6_.

Figure S4. ^13^C-NMR spectrum (100 MHz) of [Ru(2,2′-bipyridine)_2_(Cl)_2_] in DMSO-d_6_.

Figure S5. ^1^H-NMR spectrum (400 MHz) of (*E,E′*)-4,4′-Bis[p-methoxystyryl]-2,2′-bipyridine in DCM-d_2_.

Figure S6. ^13^C-NMR spectrum (100 MHz) of (*E,E′*)-4,4′-Bis[p-methoxystyryl]-2,2′-bipyridine in DCM-d_2_.

Figure S7. ^1^H-NMR spectrum (400 MHz) of [Ru(2,2′-bipyridine)_2_((*E,E′*)-4,4′-Bis[p-methoxystyryl]-2,2′-bipyridine)][Cl]_2_ in ACN-d_3_.


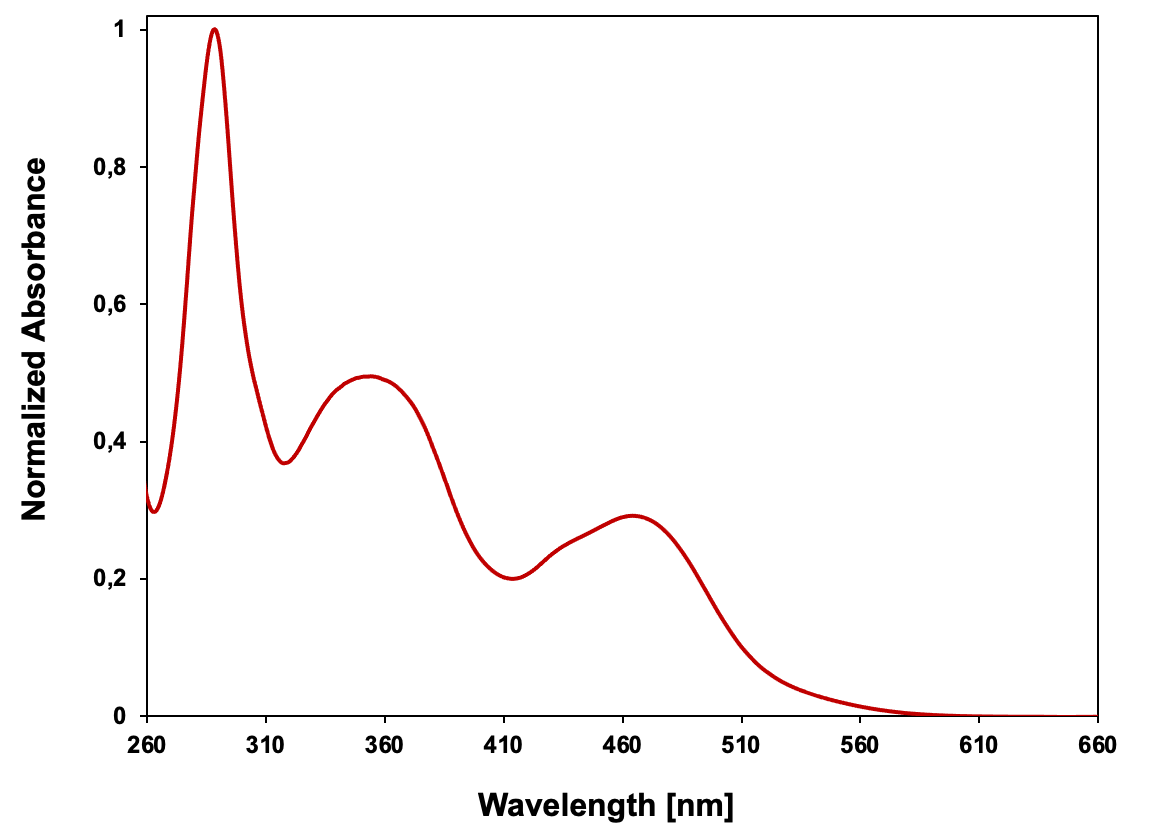


Figure S8. Normalized absorption spectra of Ru in water.


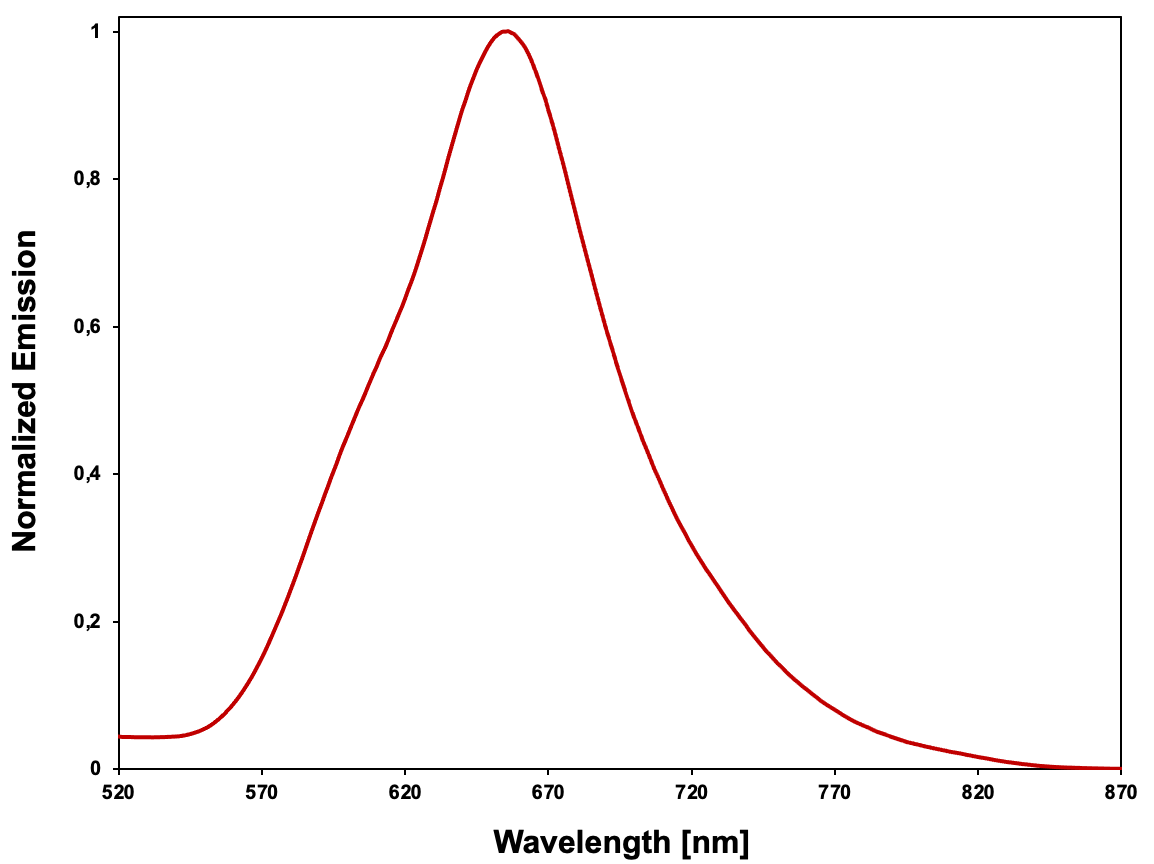


Figure S9. Normalized emission spectra of Ru in water.
